# Crystal structure and biochemical analysis of acetylesterase (*Lg*EstI) from *Lactococcus garvieae*

**DOI:** 10.1371/journal.pone.0280988

**Published:** 2023-02-06

**Authors:** Hackwon Do, Wanki Yoo, Ying Wang, Yewon Nam, Seung Chul Shin, Han-Woo Kim, Kyeong Kyu Kim, Jun Hyuck Lee

**Affiliations:** 1 Research Unit of Cryogenic Novel Material, Korea Polar Research Institute, Incheon, Korea; 2 Department of Polar Sciences, University of Science and Technology, Incheon, Korea; 3 Department of Precision Medicine, Graduate School of Basic Medical Science (GSBMS), Sungkyunkwan University School of Medicine, Suwon, Korea; 4 Department of Chemistry, Graduate School of General Studies, Sookmyung Women’s University, Seoul, Korea; 5 Division of Life Sciences, Korea Polar Research Institute, Incheon, Korea; Yale University School of Medicine, UNITED STATES

## Abstract

Esterase, a member of the serine hydrolase family, catalyzes the cleavage and formation of ester bonds with high regio- and stereospecificity, making them attractive biocatalysts for the synthesis of optically pure molecules. In this study, we performed an in-depth biochemical and structural characterization of a novel microbial acetylesterase, *Lg*EstI, from the bacterial fish pathogen *Lactococcus garvieae*. The dimeric *Lg*EstI displayed substrate preference for the short acyl chain of p-nitrophenyl esters and exhibited increased activity with F207A mutation. Comparative analysis with other esterases indicated that *Lg*EstI has a narrow and shallow active site that may exhibit substrate specificity to short acyl chains. Unlike other esterases, *Lg*EstI contains bulky residues such as Trp89, Phe194, and Trp217, which block the acyl chain channel. Furthermore, immobilized *Lg*EstI retained approximately 90% of its initial activity, indicating its potential in industrial applications. This study expands our understanding of *Lg*EstI and proposes novel ideas for improving its catalytic efficiency and substrate specificity for various applications.

## Introduction

Hydrolases catalyze the hydrolysis of a wide range of substrates, including peptides, amides, halides, esters, triglycerides, and non-natural substrates. They perform various biological functions [1, 2]. Among the α/β hydrolase enzymes, esterases catalyze the hydrolysis of a wide range of substrates with ester groups and are useful in the field of biotechnology for food processing, agriculture, detergents, and synthesis of optically active drugs [3]. Carboxylesterase from *Ochrobactrum anthropi* YZ-1, *Sulfolobus tokadaii* (EstSt7), and *Pseudomonas nitroreducens* strain CW7 exhibited catalytic activity for the detoxification of pyrethroid, which is widely used as an insecticide in agriculture and home applications and is toxic to humans [4–6]. Esterase DmtH from *Sphingobium* sp. C3 transforms dimethyl terephthalate (DMT) into the less toxic mono-methyl terephthalate (MMT). DMT is an additive widely used in the synthesis of polyester fibers, films, paints, and industrial plastics, such as polyethylene terephthalate (PET), polytrimethylene terephthalate (PTT), and polybutylene terephthalate (PBT) [7–9]. The esterase from *Bacillus subtilis* strain 1D has the potential to degrade pesticides, such as cypermethrin, in contaminated soil and water [10].

Biochemical and structural investigations of numerous esterases have been conducted to understand their biological functions [3]; common characteristics in sequences and structures are observed. The common features of esterases include canonical catalytic triad of Ser-Asp/Glu-His, conserved catalytic elbow, the HGGG motif for the stabilization of the oxyanion hole during hydrolysis [11], and several other conserved residues [12]. These features are often used for enzyme classification, predicting biological functions, and in protein engineering of esterase [13–15]. However, their systematic classification based on similarities in their primary, secondary, and tertiary structures does not always correlate with their EC numbers because of their structural plasticity and complexity [14–16]. This indicates that a detailed structural and mechanistic characterization of individual enzymes is required.

In this study, we investigated acetylesterase (*Lg*EstI) from *Lactococcus garvieae*, a major fish pathogen. We characterized the enzymatic features of *Lg*EstI that contributes to its narrow substrate specificity for acetate esters. Substrate specificity was further analyzed using three-dimensional structures, comparative analysis, and single mutagenesis of the binding pocket residues of *Lg*EstI.

## Materials and methods

### Materials

The *p*-nitrophenyl acyl esters (acetate, butyrate, octanoate, decanoate, myristate, and palmitate) were obtained from Sigma-Aldrich (Yongin, Korea). All enzymes, including Taq polymerase, thrombin, and gel filtration calibration kits for size exclusion chromatography, were obtained from Sigma-Aldrich (Yongin, Korea). Isopropyl β-d-1-thiogalactopyranoside (IPTG) and kanamycin were purchased from Fisher Scientific (Seoul, Korea).

### Protein expression and purification

Amplification of the gene encoding *Lg*EstI (NCBI accession number: WP_042219410.1) from genomic DNA was performed using PCR. The gene was cloned into the pET-28a expression vector. Competent *E*. *coli* BL21(DE3) cells were transformed with the cloned vector. Cells were grown at 37°C in lysogeny broth medium until an optical density at 600 nm (OD_600_) of 0.7 was reached. Cells were treated with 0.5 mM IPTG to induce protein expression and incubated overnight at 25°C. Cells expressing *Lg*EstI were collected by centrifugation and resuspended in NPI-20 (50 mM Tris-HCl pH 8.0, 300 mM NaCl, and 20 mM imidazole). The harvested cells were disrupted using sonication, and the soluble fractions were separated by centrifugation at 29,000 x *g* for 1 h. A two-step protein purification process was used. First, the soluble fraction was loaded onto a pre-equilibrated Ni-NTA column (Qiagen, Seoul, Korea), and the unbound debris was washed with NPI-20 (50 mM Tris-HCl pH 8.0, 300 mM NaCl, and 20 mM imidazole). *Lg*EstI bound to the resin was eluted with NPI-300 (50 mM Tris-HCl pH 8.0, 300 mM NaCl, and 300 mM imidazole). The elute was concentrated to 5 mL, and the His-tag was cleaved by incubation with thrombin for 2 days in a rotating incubator at 4°C. Following His-tag cleavage, size exclusion chromatography (SEC) was performed using HiLoad 16/600 Superdex 200 pg (GE Healthcare, Seoul, South Korea, catalog no. 28989335). The *Lg*EstI protein eluted from the SEC was collected, pooled, and concentrated to 25 mg/mL for enzyme assays and crystallization.

### Measurement of esterase activity

The influence of acyl carbon side chains of varying lengths on *Lg*EstI activity was determined by monitoring the production of *p*-nitrophenol (*p*NP), as previously described, with minor modifications [17]. Substrates were dissolved in acetonitrile. The reactions were initiated by adding substrates (250 μM) to reaction solutions containing 10 μg of the enzyme in 50 mM Tris-HCl (pH 8.0) and 200 mM NaCl. After 3 min incubation at 20°C, the production of *p*NP was determined by measuring the absorbance at 405 nm. All experiments were performed in triplicates, starting from cell cultures.

### Circular Dichroism (CD) measurement

The far-UV CD spectra were recorded with various denaturants using a spectropolarimeter (Chirascan™, Applied Photophysics Ltd., Leatherhead, UK) with *Lg*EstI at a concentration of 0.3 mg/ml in 50 mM Tris-HCl (pH 8.0) and 200 mM NaCl buffer at 20°C. The spectrum, which ranged from 200 to 250 nm, was captured using a setup with 1 nm bandwidth and 0.1 cm path length cell. To eliminate the buffer or solvent affect, the baseline spectrum of each sample was subtracted, and the five spectra were averaged. The CD data were expressed as mean residual ellipticity in deg cm^2^·dmol^-1^. The spectrum was subjected to secondary structure analysis using K2D3 online software [18].

### Generation of mutants

Site-directed mutagenesis was employed to mutate the residues F207, L208, F216, and L258, according to the manufacturer’s instructions (Agilent Technologies). Briefly, the long PCR product containing the desired sequence alteration was incubated with DPN1 to cleave the methylated template DNA and then used to transform an *Escherichia coli* host strain. The mutation sequence was confirmed through sequencing. Primers used for mutagenesis are shown in the Supporting Information.

### Crystallization and data collection and refinement

Crystallographic analysis of *Lg*EstI was conducted, as previously described [19]. The sitting-drop vapor diffusion method was used to crystallize *Lg*EstI. The *Lg*EstI crystals grew in a solution containing 0.1 M Tris: HCl, (pH 7.0), 0.2 M calcium acetate hydrate, and 20% (w/v) PEG 3000 at room temperature after several days. A single flat crystal was used to collect diffraction data with an oscillation range of 1° at beamline 5C at the Pohang Light Source-II (PLS), Korea. Native data diffracting to 2.0 Å was collected, integrated, and processed using XDS [20]. The phase was solved by molecular replacement [21] using the model from the I-TASSER [22]. The coordinates of *Lg*EstI were built and refined using a combination of Coot [23], Autobuild, and the phenix.refine package from Phenix [24, 25]. The quality of the final model was verified using a *MolProbity* [26]. The final model contained three chains in the asymmetric unit, with 99.32% residues in the favored regions of the Ramachandran plot and 0.68% residues in the disallowed region. The mutant protein, F207A, was crystallized in a buffer containing 0.2 M potassium formate (pH 7.3) and 20% (w/v) PEG 3350. A similar procedure was used to determine the structure of the F207A mutant. The coordinates and structural factors of *Lg*EstI and F207A were deposited in the Protein Data Bank RCSB under accession codes 7YC0 and 7YC4, respectively [27]. Selected X-ray data collection, phasing, and refinement statistics are presented in S1 Table. All structure-related figures were prepared using PyMOL [28].

### SEC

A Superdex 200 10/300 GL SEC column connected with ÄKTA Avant (Cytiva Marlborough, MA, USA) was prepared with a buffer containing 20 mM Tris-HCl (pH 8.0), 200 mM NaCl, 1 mM TCEP, and 5% glycerol. A sample volume of 500 μl was loaded onto the column, and the protein profile measured at 280 nm (A280) was monitored using column chromatography. Chromatography was repeated under the same conditions using a protein standard mix of 15–600 kDa (Sigma-Aldrich, cat. No. 69385-30MG) to generate a standard curve.

### Immobilizations of *Lg*EstI

To prepare cross-linked enzyme aggregates of *Lg*EstI (*Lg*EstI-CLEAs), 1 mg/ml of *Lg*EstI was precipitated with 80% ammonium sulfate and 10 mM of glutaraldehyde in 50 mM PBS. After overnight incubation at room temperature, *Lg*EstI-CLEAs were collected by centrifugation at 15,000 × g for 10 min. *Lg*EstI-CLEAs were then washed and stored at 4°C until use. The enzymatic activity of *Lg*EstI-CLEAs was investigated using *p*NA as a substrate. *Lg*EstI-CLEAs were incubated in 500 μL of 250 μM *p*NA solution for 3 min. The reaction mixture was centrifuged for 5 min, following which the absorbance of the supernatant was measured at 405 nm. To examine the reusability of *Lg*EstI-CLEAs, the pellet was washed with buffer until no activity was detected. The reaction was then repeated with a new substrate. All experiments were performed in triplicates, starting from cell cultures.

## Results and discussion

### Biochemical characterization of *Lg*EstI

**Fig 1:**
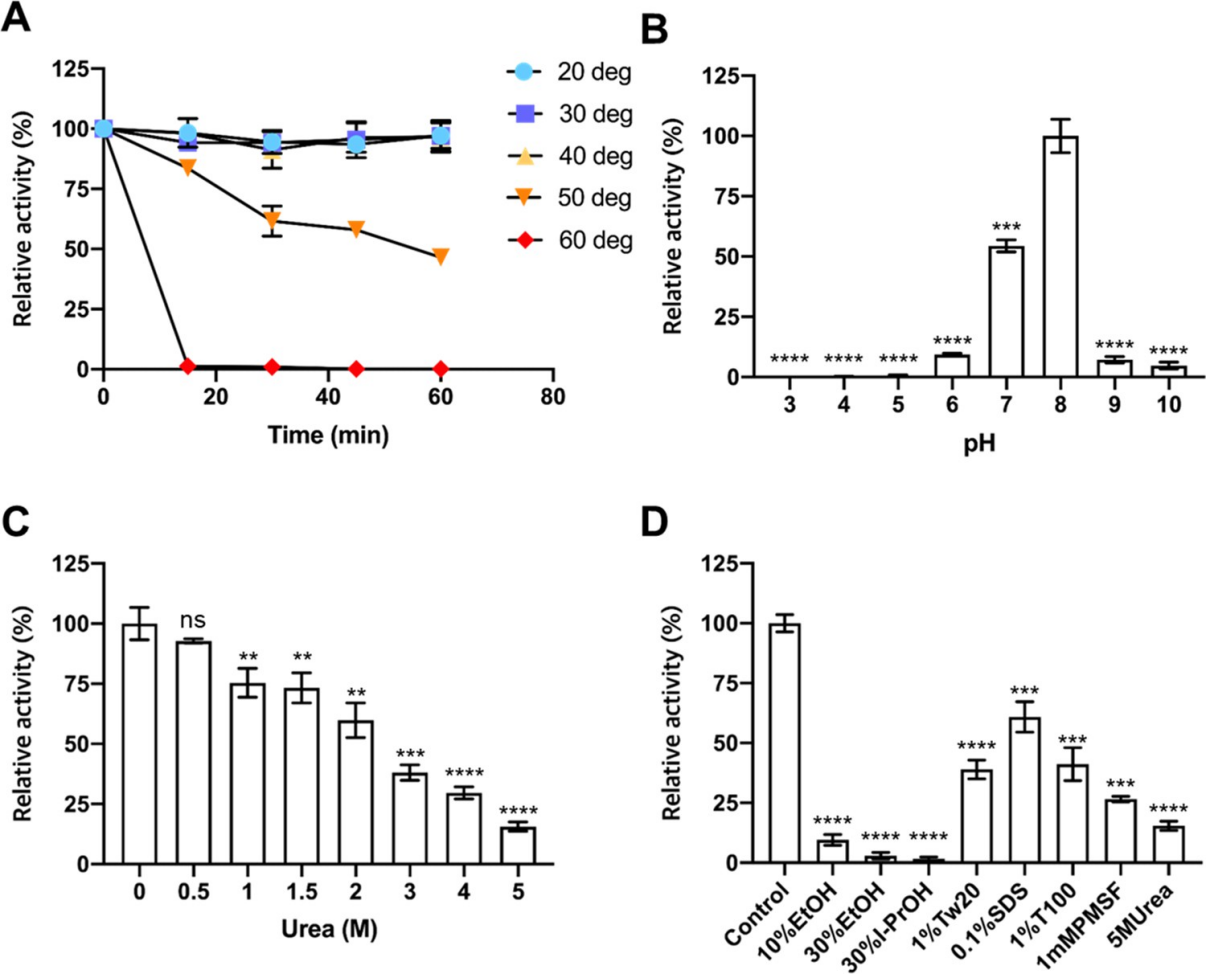
Characterization of the esterase *Lg*EstI. (A) The effect of temperature on enzyme activity. Enzyme activity was measured at a temperature range of 20–60°C every 15 min for 1 h. The activity value obtained at 20°C was defined as 100% (refer to S2 Table). (B) The effect of pH on enzyme activity. Enzyme activity was determined in citrate buffer (pH 3.0–5.0), phosphate buffer (pH 6.0–7.0), Tris-HCl buffer (pH 8.0), and glycine buffer (pH 9.0–10.0) at 25°C. The activity at pH 8.0 using *p*NA was defined as 100%. The activity at pH 8.0 was used as a reference and percentage levels at each pH relative to the reference are shown. (C and D) Effect of organic solvents on *Lg*EstI activity in the presence of *p*NA. Residual activity of *Lg*EstI after incubating for 1 h with the indicated chemicals. Activity without the addition of chemical compounds was defined as 100% and relative activity was expressed. All data are presented as mean ± standard deviation (s.d.) (n = 3). The activity without denaturant was used as the reference and percentage levels relative to the reference are shown. Statistical significance (*p*-values) was calculated using a *t*-test. p values were indicated by ns, not significant (p > 0.05), * (p < 0.05), ** (p < 0.01), *** (p < 0.001), and **** (p < 0.0001). Statistical analyses were performed in GraphPad Prism software v.8.3.0.

*Lg*EstI prefers the short acyl chain of *p*-nitrophenyl esters [19]. When different *p*-nitrophenyl esters with the acyl carbon group ranging from C_2_ to C_12_ were tested, only *p*NA was effectively cleaved by *Lg*EstI. Moreover, *Lg*EstI exhibited decreased activity (less than 10%) for *p*NH and *p*NO, and no activity against other substrates [19]. The thermostability of *Lg*EstI was tested every 15 min for 1 h at temperatures ranging from 20–60°C, revealing that the activity of *Lg*EstI was not significantly altered until 40°C; however, the activity was lowered to 50% after 60 min at 50°C (Fig 1A). Furthermore, when tested against *p*NA at various pHs, *Lg*EstI activity was the highest at pH 8.0 (Fig 1B).

In industrial applications, esterases can withstand unfavorable conditions, such as high concentrations of alcohol, detergent, and chemicals that interact with the protein. We further examined the chemical stability of *Lg*EstI against various denaturants by checking its activity and changes in secondary structures. The results indicated a reduction in activity when exposed to urea, at 0.5 to 5 M, in a concentration-dependent manner (Fig 1C). Similarly, far-UV CD spectroscopy results indicated urea-induced unfolding transition (S3 Fig). The spectrum change was initiated from below 210 nm; 3M urea almost completely denatured the helix region in *Lg*EstI, indicating that urea denatures *Lg*EstI by promoting backbone disorder in the helix region. In addition, *Lg*EstI was susceptible to unfolding by alcohol (10% and 30%) and isopropyl alcohol (30%) (S3 Fig). Considering the Urea-concentration dependent decrease in activity and the fact that the cap domain of esterases consists only of helices, which are responsible for enzyme activity, substrate binding, thermophilicity, and thermostability [29], these findings suggest that the cap domain of *Lg*EstI could be the initial target in the unfolding by denaturants.

### Overall structure of *Lg*EstI

To better understand the biochemical properties of *Lg*EstI, structural determination and comparative analyses were performed. The three-dimensional structure of *Lg*EstI was determined using X-ray crystallography at 2.0 Å resolution. First, we generated the *Lg*EstI model based on homologous structures. The quality of the model was confirmed with the 1.40 and 0.91±0.06 C-score and TM-score, respectively. The *Lg*EstI model was used to solve the phase problem using the molecular replacement method. After iterative model building and refinement, the final structure was refined to R_work_ and R_free_ values of 0.19 and 0.23, respectively (S1 Table). Most residues were well fitted to the electron density maps, with a *MolProbity* score of 1.50 [26]. An *Lg*EstI crystal contained three monomers per asymmetric unit. Three cis peptides were found at identical locations (His21–Pro22, Ser120–Pro121, and Tyr125–Pro126) in the three monomers. Superposition analysis revealed that each monomer was almost identical, with a root-mean-square deviation (RMSD) of ~ 0.3 Å (Cα), including most of the water molecules surrounding the monomers. Therefore, chain A was used for further structural description and analysis.

**Fig 2:**
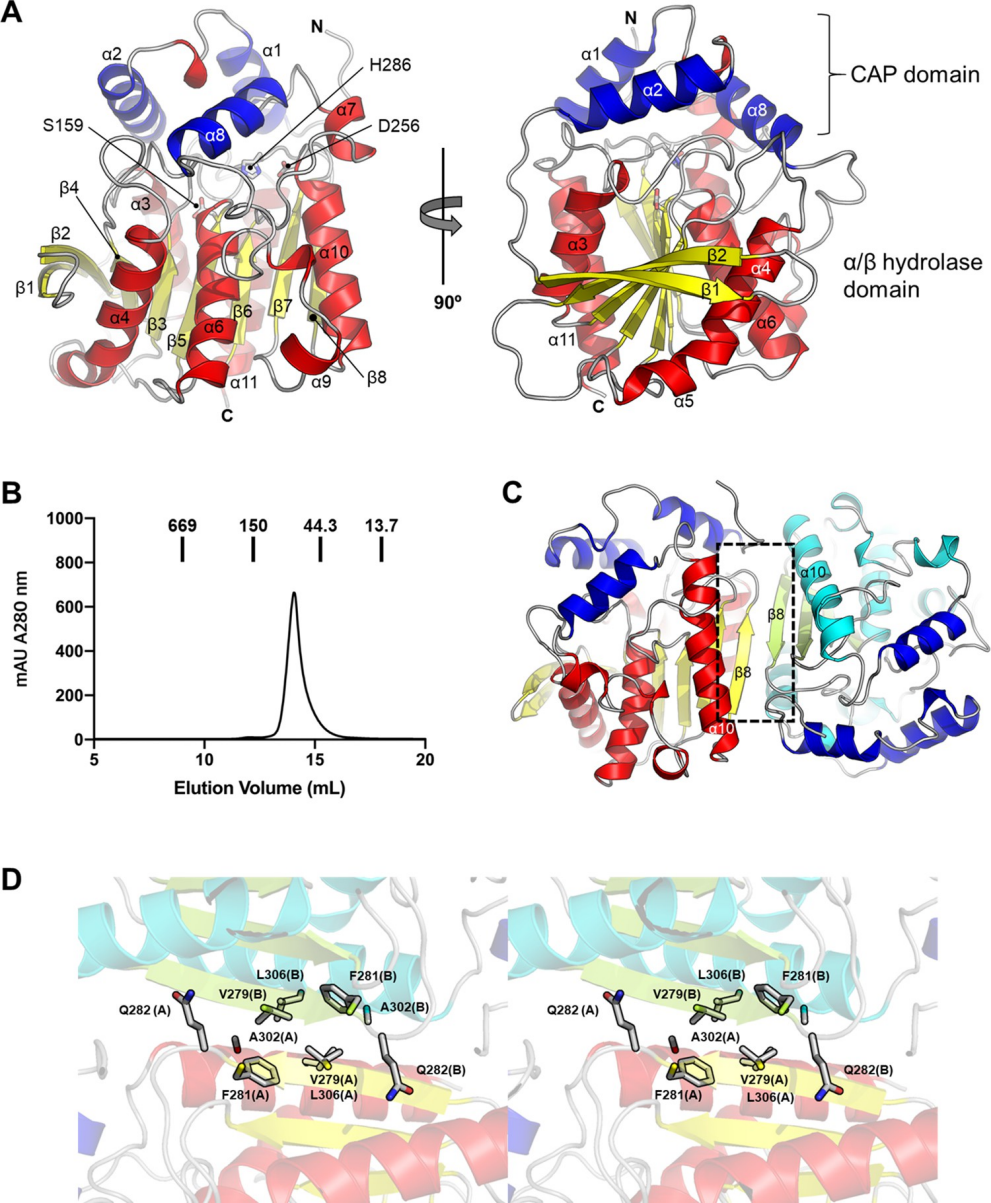
Overall structure of *Lg*EstI and dimer state. (A) Cartoon representation of the *Lg*EstI monomer with a front view (left) and 90° rotated view (right). The CAP domain made up of three triangular helices; α1, α2, and α8 are shown with blue, while the α/β hydrolase domain is indicated with red for helices and yellow for strains. The residues of the catalytic triad (Ser159, Asp256, and His286) are represented by a stick. (B) Size exclusion chromatography (SEC) profile of *Lg*EstI. Measured molecular mass of *Lg*EstI was 70.91 kDa, which is close to the sequence-based molecular weight of the dimer (72.05 kDa). (C) Overall dimer presentation of *Lg*EstI and the dimer interface is indicated with a dotted square. (D) Stereo view of the dimer interface of *Lg*EstI. Residues involved in the dimeric interface of *Lg*EstI are represented with sticks. The chains are differentiated with color: red for chain A and cyan for chain B, and marked after the residue number with parentheses.

Monomeric *Lg*EstI exhibits a canonical α/β hydrolase fold with conserved key active site residues. The monomer can be divided into two domains: a CAP domain and an α/β hydrolase domain. The CAP domain is made up of three triangular helices, α1, α2, and α8, whereas the α/β hydrolase domain consists of a 90-degree twisted β sheet in the center, five neighboring α-helices (α3, α4, α6, α10, and α11), and turns connecting helices and strands (Fig 2A). SEC revealed that *Lg*EstI formed a dimer in solution (Fig 2B and S1 Fig). The dimer interface was associated with a central hydrophobic patch consisting of α10, α11, and β8. Val279 and Phe281 from β8, and Ala302 and Leu306 from α11 of chains A and B contribute to the dimer formation. Specifically, the two Gln282 residues from each chain are deeply involved in multiple hydrogen bonds, making this interface stronger and broader as 1087.9 Å^2^ of the averaged-buried area (Fig 2C and 2D). This dimeric assembly is the dominant pattern of esterase/lipase; however, few other orthologs have distinct oligomeric states with similar sequences and structural similarities [30].

### Substrate access channel of *Lg*EstI

**Fig 3:**
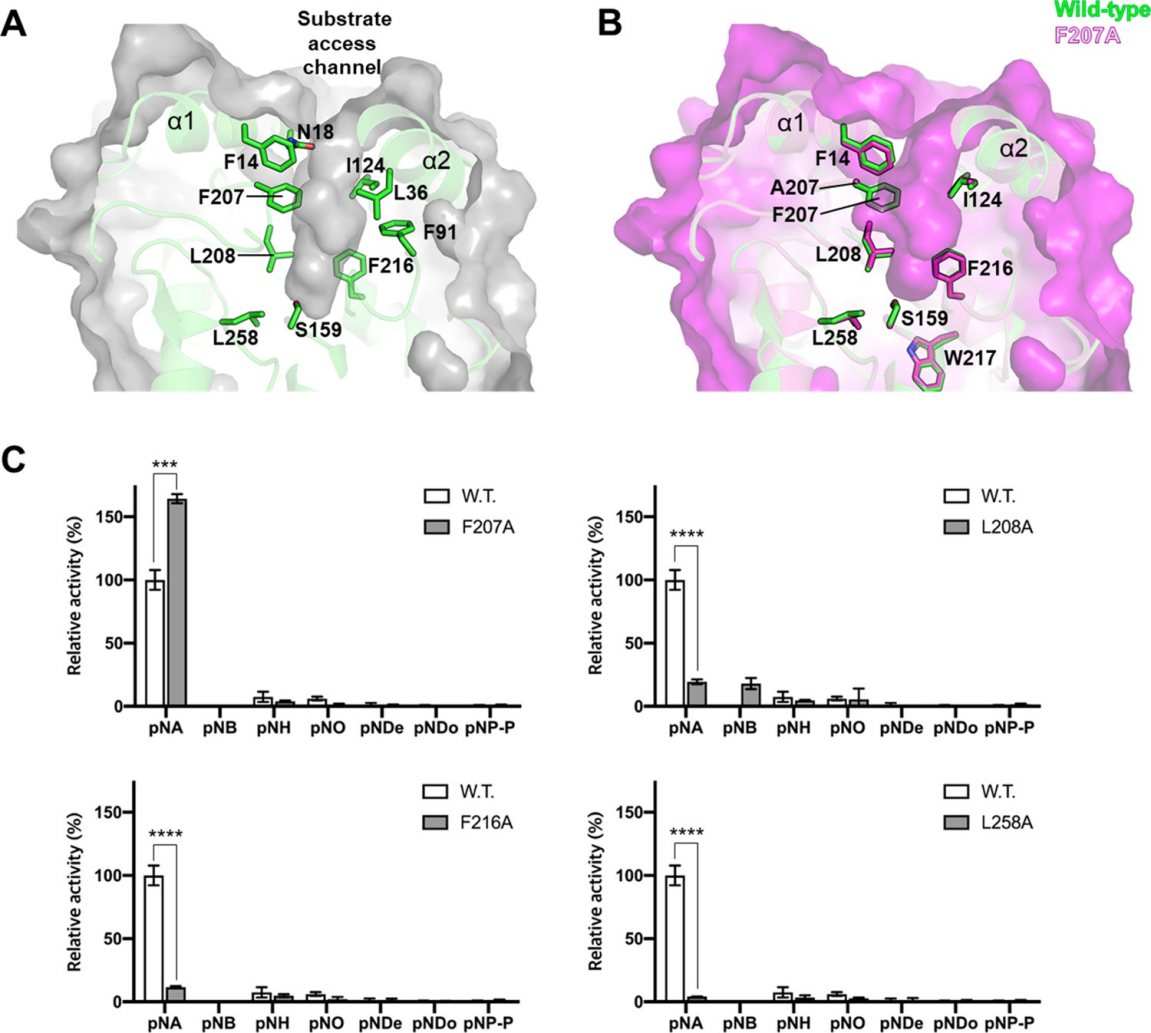
Characterization of the active site of *Lg*EstI. (A) Representation of the *Lg*EstI substrate access channel. Illustrated and surface views of the enzyme. (B) Superimposed structure of *Lg*EstI wild-type (green) and mutant F207A (magenta). Residues around the substrate access channel are depicted with sticks. (C) Comparative activity of the wild-type *Lg*EstI and mutant enzymes with various substrates. The activity value obtained with wild-type *Lg*EstI was defined as 100%. All data are presented as mean ± standard deviation (s.d.) (n = 3). The activity of *Lg*EstI wild-type against *p*NA was used as the reference and percentage levels to the reference are shown. Statistical significance (*p* values) was calculated using a *t*-test. P values were indicated by *** (p < 0.001), and **** (p < 0.0001). Statistical analyses were performed in GraphPad Prism software v.8.3.0.

Cavity analysis and structural comparison of *Lg*EstI with known orthologs indicated that the substrate access channel to the active site of *Lg*EstI was relatively narrow and shallow. The funnel-like channel was approximately 16 Å deep (between CD2 of Leu35 and OG of Ser159) and 6.5 Å wide (between CZ of Phe207 and CD1 of Ile24). The wall comprising the substrate access channel was surrounded by Phe14, Asn18, Ile24, Lue36, Gly88, Phe91, Phe207, Lue208, Met213, Phe216, Lue258, and Met290 (Fig 3A). Among these residues, Phe207, Lue208, Phe216, and Lue258 were in proximity to the catalytic triad; they probably recognize *p*-nitrophenyl ester and interact with it through hydrophobic interactions. To investigate the role of each residue on activity and substrate specificity, Phe207, Lue208, Phe216, and Lue258 were mutated to alanine through site-directed mutagenesis (S2 Fig). The catalytic activity was examined with various substrates and compared with that of the wild-type. The activity of all variants against *p*NA was lower than that of wild-type *Lg*EstI. However, F207A exhibited a significantly increased hydrolytic activity (over 150%) toward *p*NA. In addition, L208A exhibited slightly increased activity against *p*NB, which was not observed in the wild-type, F207A, F216A, and L258A strains (Fig 3C). Decreased activity with a single amino acid mutation around the active site indicates that the elaborate spatial composition of the active site environment is as important as the catalytic triad for proper catalysis and substrate orientation [31].

To understand the increased activity of F207A against *p*NA at the molecular level, its structure was determined at a resolution of 2.1 Å. The overall structure of F207A was almost identical to that of wild-type *Lg*EstI, with 0.177 of RMSD (Cα of monomer); in addition, the oligomerization form remained unchanged (S1 Fig). No substantial movement around the active site was observed, implying that there could be multiple interactions among the residues that secure the interior conformation. However, Phe14 was shifted by approximately 0.5 Å due to the spatial reduction caused by the mutation of Phe207 to alanine. Phe14 in the wild-type protein interacted with Phe207 via edge-face interactions and was oriented toward the substrate access channel. The additional space created by the disappearance of Phe207 was partially occupied by Phe14 (Fig 3B). Another notable difference is the increased area for the substrate, a consequence of the mutation. The pocket volume based on the molecular surface (MS) volume increased from 548.4 Å^3^ to 657.8 Å^3^ in the F207A structure (S3 Table). Therefore, area expansion could increase substrate accessibility and/or dissociation of the product, thereby increasing enzyme activity.

**Fig 4:**
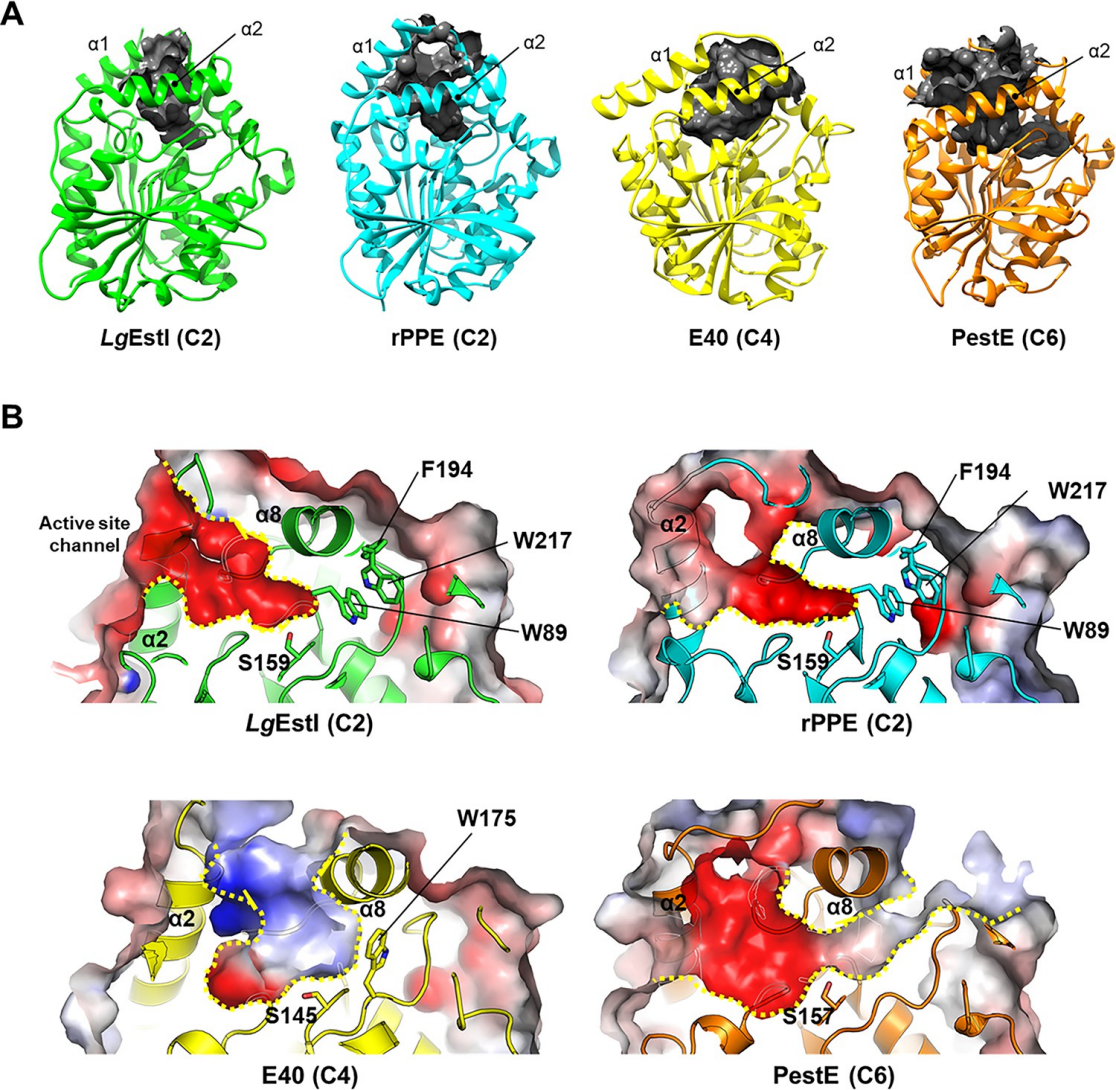
Comparison of the substrate access channel. (A) The surface area and volume of esterase and lipase based on the molecular surface are represented with black surfaces. The Computed Atlas of Surface Topography of proteins (CASTp) was employed to determine protein volume (probe radius of 1.4 Å). (B) Electrostatic potential surface and diagrammatic representation of the substrate access channel of the enzymes. The surface charge distribution is displayed as blue for positive, red for negative, and white for neutral. The channel of each enzyme is highlighted with yellow dots. Bulky residues blocking the channel of *Lg*EstI, rPPE, and E40 are indicated with sticks.

The F207 mutation increased activity against *p*NA; however, F207A did not exhibit increased activity against other substrates with long acyl chains in parallel, when compared to *Lg*EstI (Fig 3C), indicating that other factors could be involved in substrate selectivity. Structural comparisons with orthologs indicated that esterases have various tunnel configurations (Table 1). The pocket volume of *Lg*EstI (C2) is 548.4 Å^3^, while that of E40 (C4) [32] and PestE (C6) [33] based on the MS are 1100.8 Å^3^ and 1934 Å^3^, respectively (Fig 4A). These differences in volume originate from the size of the mouth area and length of the tunnel for the acyl chain. PestE (C6) has a long tunnel located beneath α8 for the carbon chain that links the substrate entrance to the outer surface [33]. Ser157 is located in the middle of a narrow tunnel for substrate cleavage. However, *Lg*EstI and rPPE (C2) have shallow substrate tunnels. When compared with PestE (C6), Trp89, Phe194, and Trp217 of *Lg*EstI and rPPE protrude into the tunnel. Therefore, the tunnel observed in PestE is blocked by bulky residues in *Lg*EstI and rPPE. Tunnel blocking by a bulky residue (Trp175) is also observed in E40 (C4) (Fig 4B). Therefore, the limited space available for *Lg*EstI may hinder the access of substrates with long acyl carbon chains. Area expansion of the acyl binding pocket located at the bottom of the active site changes the substrate preference of rPPE [31]. Structural and activity analyses of rPPE revealed that the mutation of W187H increased the volume of the acyl chain pocket and changed the substrate preference from short *p*NA to pNB, which has a long acyl carbon chain [31, 34]. In conclusion, *Lg*EstI exhibits substrate specificity against *p*NA owing to its narrow binding site and blocked tunnel, which favors short acyl chains. Structural comparison indicated that the cavity channel of the esterase/lipase was wider and longer as the length of the substrate acyl chain increased.

**Table 1:** Structural homolog search result for *Lg*EstI from a DALI search (DALI-Lite server).

Protein	PDB code	DALI Z-score	UniProtKB code	Sequence % ID with *Lg*EstI (aligned residue number)	Reference
rPPE	4OB8	49.4	L7PYQ2	41 (313/319)	[31]
ThaEst2349	4V2I	49.1	A0A023T3X2	47 (313/315)	[35]
CinB	6KMO	48.1	N/A	35 (311/330)	[36]
*Rm*EstB	4WY8	46.5	A0A0M3KKZ9	39 (311/325)	[37]
Est8	4YPV	46.0	P29575	37 (306/313)	[38]
*Rm*EstA	4WY5	44.0	V5J5W4	35 (310/320)	[37]
TmelEST2	5MII	43.8	D5GA36	28 (306/307)	[39]
EstA	5LK6	43.3	F0NDQ1	33 (299/302)	Not published
PestE	3ZWQ	42.6	A3MVR4	34 (303/313)	[33]
EST2	1QZ3	42.1	Q7SIG1	34 (302/309)	[40]

N/A, not available

### Substrate specificity of *Lg*EstI

**Fig 5:**
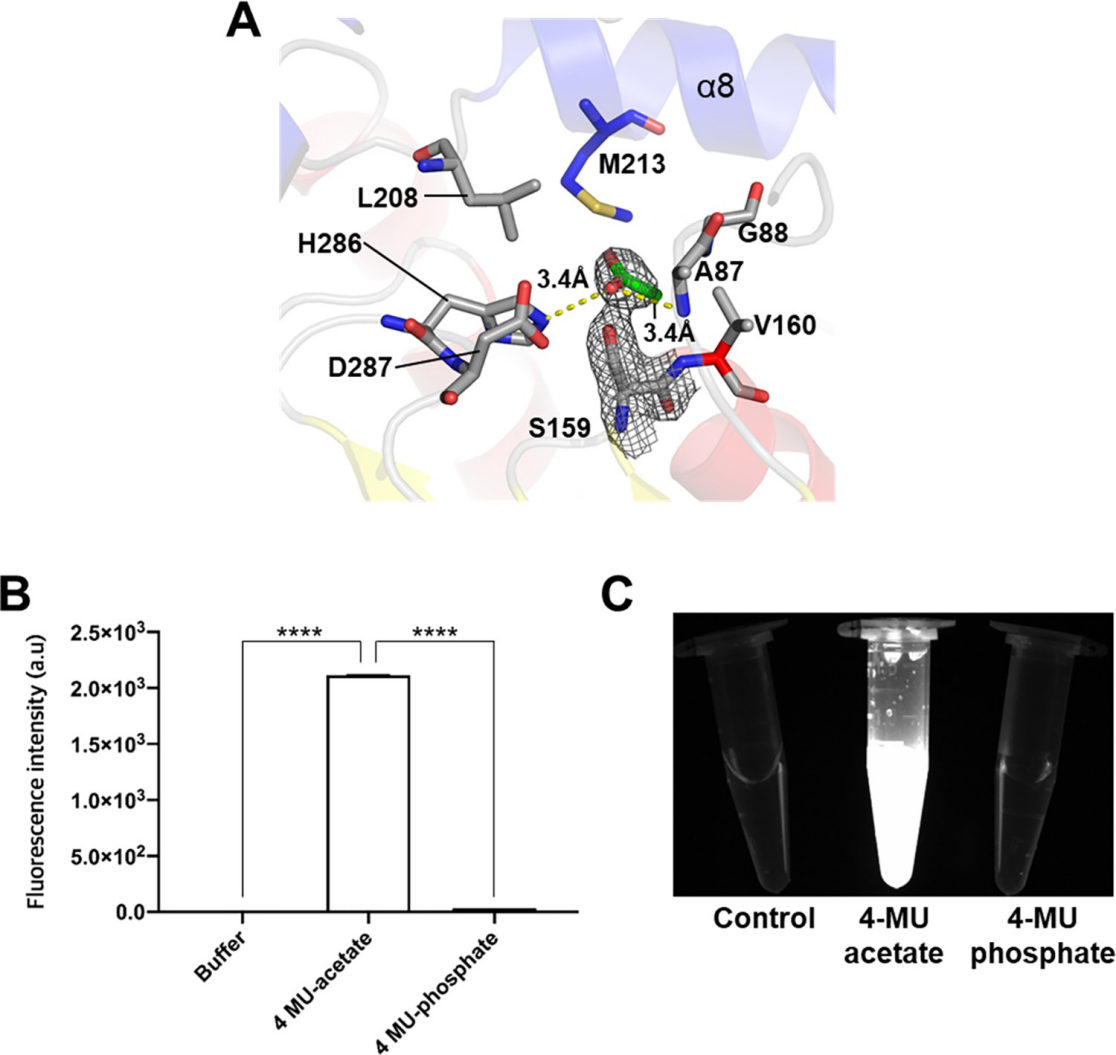
Active site and substrate specificity of *Lg*EstI. (A) A close-up view of the active site illustrating the coordination between the active site residues and the acetate moiety connected to nucleophilic S159. Hydrolysis of 4-methylumbelliferyl (4-MU) acetate and phosphate by *Lg*EstI was measured (B) and observed (C). Control indicates *Lg*EstI only. All data are presented as mean ± standard deviation (s.d.) (n = 3). Statistical significance (*p* values) was calculated using a *t*-test. P values were indicated by ns, not significant (P > 0.05), * (p < 0.05), ** (p < 0.01), *** (p < 0.001), and **** (p < 0.0001). Statistical analyses were performed in GraphPad Prism software v.8.3.0.

The active site of *Lg*EstI has the typical catalytic triad of esterases, identified as Ser159, Asp256, and His286, and is located at the bottom of the substrate access channel. Careful investigation of the active site of wild-type *Lg*EstI indicated that two of the three monomers had a triangular blob at the tip of the hydroxyl group of Ser159. The crystallization condition included 0.1 M calcium acetate hydrate; therefore, we incorporated acetate into the blob as it had a 0.5 reasonable density fit value [23]. Another possibility is a residual product of hydrolytic activity. The acetate moiety was positioned at the bottom of the active site and surrounded by Val160, Met213, His286, and Asp287 within hydrogen bonding distances, and an oxyanion hole consisting of Ala87 and Gly88. The oxygen atom interacts with the NE2 of His286, and the methyl group of acetate is directed toward Val160 for hydrophobic interactions (Fig 5A). To determine whether *Lg*EstI is specific to acetylated substrates, we measured its activity using fluorogenic substrates: 4-Methylumbelliferone (MU) acetate and 4-Methylumbelliferone (MU) phosphate, which have similar overall structures but different tails at O13. *Lg*EstI was active only against 4-MU acetate (Fig 5B and 5C). In addition to the length of the acyl chain, the acetate moiety of the substrate is a critical factor for substrate recognition of *Lg*EstI.

### Immobilization of *Lg*EstI

**Fig 6:**
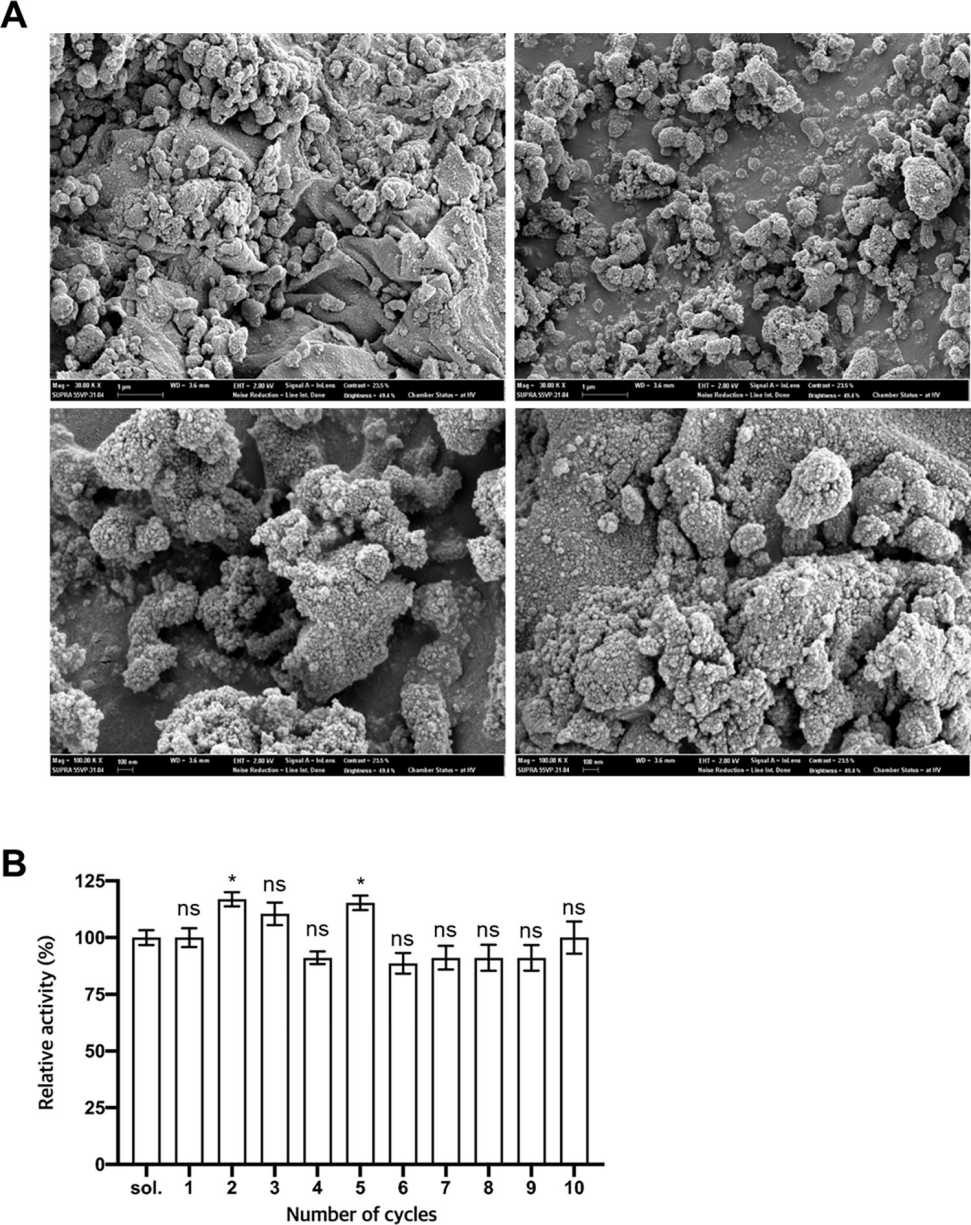
Immobilization of *Lg*EstI and activity. (A) Scanning electron microscope (SEM) image of *Lg*EstI-CLEAs. Representative images at 30 kX (upper) and 100 kX (lower) are shown. (B) The reusability of *Lg*EstI-CLEAs was assessed by measuring residual activity after repeated washing. *p*NA was used as a substrate and the activity of soluble *Lg*EstI was defined as 100%. Statistical significance (*p* values) was calculated using a *t*-test. P values were indicated by ns, not significant (P > 0.05), and * (p < 0.05).

Esterase is a potential biocatalyst in industrial applications, and immobilization is a widely used method for ensuring the recyclability and stability of enzymes [41–44]. The formation of cross-linked enzyme aggregates (CLEAs) is often used for enhancing enzyme applicability in the industry as it enables enzyme stability and ensures enzyme reusability. Therefore, we immobilized *Lg*EstI by precipitating the protein with ammonium sulfate and glutaraldehyde to generate *Lg*EstI-CLEAs. Immobilization conditions were optimized by varying the concentration of glutaraldehyde; 10 mM glutaraldehyde, which enabled the highest enzyme activity, was chosen for the preparation of *Lg*EstI-CLEAs. *Lg*EstI-CLEA formed globe-like structures with a size of 0.2 μm with a highly ordered and rigid structure, as observed using scanning electron microscopy (SEM) (Fig 6A). The activity was comparable to that of the control (soluble form of *Lg*EstI in Fig 6B). To check reusability, the activities of *Lg*EstI-CLEA were measured between washing steps. There was no significant decrease in activity during the washing steps. The activity of *Lg*EstI-CLEAs after 10 cycles was approximately 90% of the initial activity, confirming their usability and recyclability. The immobilized form has suitable solidity, density, and porosity [45]. The esterases have been tested for the deprotection of acetyl groups from monosaccharides and β-lactam antibiotics [46, 47], and they are used in organic synthesis; therefore, *Lg*EstI could be of potent use in the food and medical fields.

## Conclusion

*In silico* analysis of the genomic DNA of *L*. *garvieae* has enabled the identification of several putative esterases/lipases [48]. Among the esterases, *Lg*EstI was characterized using biochemical and structural analyses. *Lg*EstI belongs to the HSL family of α/β-hydrolases [13] and presents a clear substrate preference for a short acyl chain and an acetate moiety that are suitable for the narrow entrance to the active site. The volume of the binding pocket for the substrate head group had a greater influence on the activity increment for a specific substrate. Substitution of the Phe207 residue allowed increased substrate access to the active site, as evidenced by increased hydrolysis activity. The substrate specificity of the esterase for a short acyl chain could be attributed to blocked residues in the substrate access tunnel. The esterases with substrate preference for a short acyl chain have bulky residues that block the tunnel for the acyl chain. *Lg*EstI can be used in an immobilized form (*Lg*EstI-CLEAs); it exhibits a stable activity for at least 10 repeated washing cycles. Therefore, the substrate specificity, high stereoselectivity, and immobilization ability of *Lg*EstI make it useful for applications pertaining to biotransformation, food additives, and detergents.

## Supporting information

S1 Table X-ray diffraction data collection and refinement statistics. (DOC)

S2 Table Kinetic parameters for hydrolysis of *p*NA by purified *Lg*EstI. (DOC)

S3 Table Surface pocket analysis of the enzymes using CASTp. (DOC)

S1 Fig Oligomeric state of *Lg*EstI. (DOC)

S2 Fig SDS-PAGE analysis of the recombinant wild-type and *Lg*EstI variants. (DOC)

S3 Fig Circular dichroism (CD) spectroscopy of *Lg*EstI with various denaturants. (DOC)
