# Supplementary material for: Crystal structure and biochemical analysis of acetylesterase (LgEstI) from Lactococcus garvieae
Source: PLoS One. 2023 Feb 6;18(2):e0280988. doi: 10.1371/journal.pone.0280988 (PMC9901739; doi:10.1371/journal.pone.0280988)
Supplement: S1 Table — (DOC) [file pone.0280988.s001.doc]

**Supplemental Table S1. X-ray diffraction data collection and refinement statistics.**

| Data set | *Lg*EstI | F207A |
| --- | --- | --- |
| X-ray source | BL-5C beamline | BL-5C beamline |
| Space group | *I*121 | *P*12_1_1 |
| Unit-cell parameters (Å, °) | a=54.41, b=92.77, c=218.11, α=γ=90.0, β=96.61 | a=99.20, b=127.39, c=104.76, α=γ=90, β=90.17 |
| Wavelength (Å) | 0.97949 | 0.97949 |
| Resolution (Å) | 28.49–2.0 (2.05–2.0) | 29.35–2.1 (2.14–2.1) |
| Total reflections | 494404 | 1021498 |
| Unique reflections | 71847 (4652) | 151387 (7500) |
| Average I/σ (I) | 16.1 (2.8) | 12.3 (3.0) |
| *R*_merge_^a^ | 0.059 (0.540) | 0.100 (0.712) |
| Redundancy | 6.9 (7.2) | 6.7 (7.2) |
| Completeness (%) | 98.7 (99.8) | 99.9 (99.9) |
| Refinement |  |  |
| Resolution range (Å) | 28.51–2.0 (2.05–2.0) | 29.35–2.1 (2.14–2.1) |
| No. of reflections of working set | 68254 (5106) | 143805 (10604) |
| No. of reflections of test set | 3591 (249) | 7581 (566) |
| No. of amino acid residues | 946 | 3094 |
| No. of water molecules | 526 | 586 |
| *R*_cryst_^b^ | 0.19 (0.23) | 0.19 (0.25) |
| *R*_free_^c^ | 0.23 (0.35) | 0.24 (0.31) |
| R.m.s. bond length (Å) | 0.008 | 0.007 |
| R.m.s. bond angle (°) | 1.570 | 1.539 |
| Average B value (Å^2^) (protein) | 41.38 | 37.21 |
| Average B value (Å^2^) (solvent) | 43.86 | 35.28 |
| Ramachandran plot |  |  |
| Favored (%) | 95.53 | 94.85 |
| Allowed (%) | 4.15 | 4.55 |
| Outliers (%) | 0.32 | 0.6 |

^a^ *R*_merge_ = ∑｜<I> - I｜/∑<I>.

^b^ *R*_cryst_ = ∑｜|Fo| - |Fc|｜/∑|Fo|.

^c^ *R*_free_ calculated with 5% of all reflections excluded from refinement stages using high-resolution data.

Values in parentheses refer to the highest-resolution shells.
