# Supplementary material for: Crystal structure and biochemical analysis of acetylesterase (LgEstI) from Lactococcus garvieae
Source: PLoS One. 2023 Feb 6;18(2):e0280988. doi: 10.1371/journal.pone.0280988 (PMC9901739; doi:10.1371/journal.pone.0280988)
Supplement: S2 Table — (DOC) [file pone.0280988.s002.doc]

**Supplemental Table S2.** Kinetic parameters for hydrolysis of *p*NA by purified *Lg*EstI.

| Substrate | *V_max_*  (nM s^-1^) | *K_m_*  (mM) | *K_cat_*  (s^-1^) | *K_cat_/K_m_*  (mM^-1^ s^-1^) |
| --- | --- | --- | --- | --- |
| *p*NA | 435.9 ± 1.8 | 1.6 ± 0.1 | 15.7 ± 0.7 | 10.8 ± 1.0 |
