# Supplementary material for: Crystal structure and biochemical analysis of acetylesterase (LgEstI) from Lactococcus garvieae
Source: PLoS One. 2023 Feb 6;18(2):e0280988. doi: 10.1371/journal.pone.0280988 (PMC9901739; doi:10.1371/journal.pone.0280988)
Supplement: S3 Table — (DOC) [file pone.0280988.s003.doc]

**Supplemental Table S3.** Surface pocket analysis of the enzymes using CASTp [1].

| Esterase | Substrate preference | MS volume^1^ | Pocket MS area^2^ | Mouth MS area^3^ | MS circumference sum^4^ | Reference |
| --- | --- | --- | --- | --- | --- | --- |
| *Lg*EstI | C2 | 548.4 | 558.4 | 44.1 | 43.0 | This work |
| F2027A | C2 | 657.8 | 578.3 | 47.0 | 40.0 | This work |
| rPPE (4OB8) | C2 | 1101.7 | 676.3 | 174.7 | 89.8 | [2] |
| E40 (4XVC) | C4 | 1100.8 | 831.0 | 99.4 | 60.8 | [3] |
| PestE (3ZWQ) | C6 | 1934.0 | 1310.8 | 183.4 | 132.8 | [4] |

^1^MS volume-pocket volume based on the molecular surface.

^2^Pocket MS area - pocket molecular surface area.

^3^Mouth MS area: total area of mouth opening(s) based on molecular surface.

^4^MS circumference sum - total circumference of mouth opening(s) based on the molecular surface.

1. Tian W, Chen C, Lei X, Zhao J, Liang J. CASTp 3.0: Computed atlas of surface topography of proteins. Nucleic Acids Res. 2018;46: W363–W367. doi:10.1093/nar/gky473

2. Dou S, Kong XD, Ma B Di, Chen Q, Zhang J, Zhou J, et al. Crystal structures of Pseudomonas putida esterase reveal the functional role of residues 187 and 287 in substrate binding and chiral recognition. Biochem Biophys Res Commun. 2014;446: 1145–1150. doi:10.1016/j.bbrc.2014.03.072

3. Li PY, Chen XL, Ji P, Li CY, Wang P, Zhang Y, et al. Interdomain hydrophobic interactions modulate the thermostability of microbial esterases from the hormone-sensitive lipase family. J Biol Chem. 2015;290: 11188–11198. doi:10.1074/jbc.M115.646182

4. Palm GJ, Fernández-Álvaro E, Bogdanović X, Bartsch S, Sczodrok J, Singh RK, et al. The crystal structure of an esterase from the hyperthermophilic microorganism Pyrobaculum calidifontis VA1 explains its enantioselectivity. Appl Microbiol Biotechnol. 2011;91: 1061–1072. doi:10.1007/s00253-011-3337-9
