# Supplementary material for: Crystal structure and biochemical analysis of acetylesterase (LgEstI) from Lactococcus garvieae
Source: PLoS One. 2023 Feb 6;18(2):e0280988. doi: 10.1371/journal.pone.0280988 (PMC9901739; doi:10.1371/journal.pone.0280988)
Supplement: S1 Fig — (DOC) [file pone.0280988.s004.doc]

**
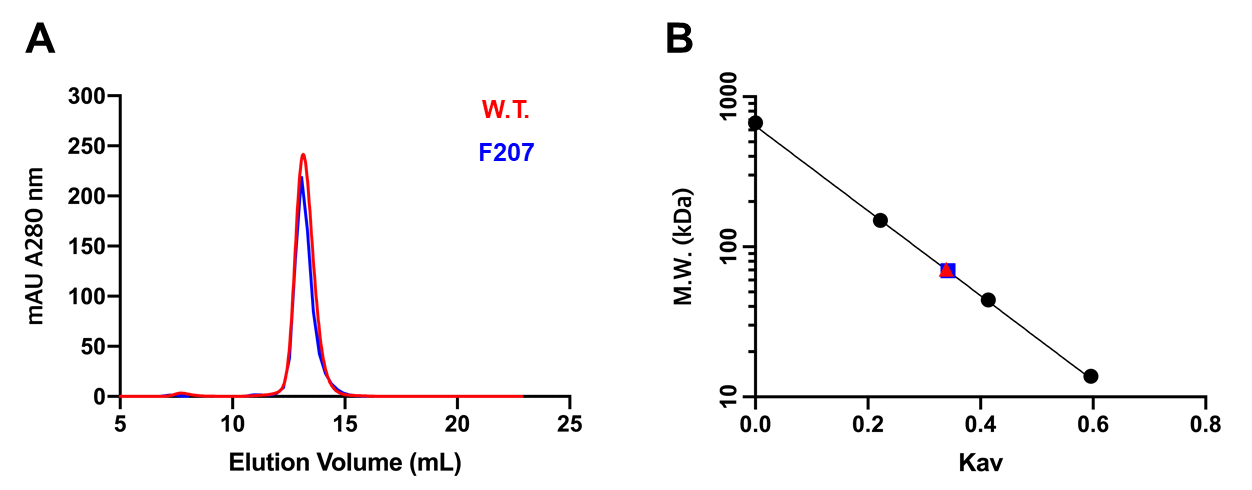
**

**Supplemental Figure S1.** The oligomeric state of LgEstI and F207A. (A) Size exclusion chromatography profiling for *Lg*EstI and F207A was performed at 280 nm. Superdex 200 10/300 GL column connected with ÄKTA Avant system (Cytiva) was used. (B) The protein standard mix ranged from 15 to 600 kDa (Sigma-Aldrich, cat. No. 69385-30MG) and was used to generate a standard curve under the same conditions. The calculated molecular mass of *Lg*EstI and F207A was 70.91 and 69.39 kDa, respectively.
