# Supplementary material for: Crystal structure and biochemical analysis of acetylesterase (LgEstI) from Lactococcus garvieae
Source: PLoS One. 2023 Feb 6;18(2):e0280988. doi: 10.1371/journal.pone.0280988 (PMC9901739; doi:10.1371/journal.pone.0280988)
Supplement: S2 Fig — (DOC) [file pone.0280988.s005.doc]

**
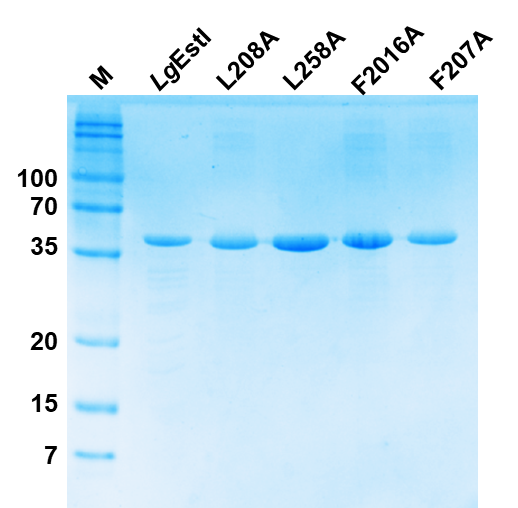
**

**Supplemental Figure S2.** SDS-PAGE analysis of the recombinant wild-type and *Lg*EstI variants. All proteins were purified using His-tag affinity and SEC, as described in the “Experimental Procedures” section. M, protein marker (kDa).
