# Supplementary material for: Crystal structure and biochemical analysis of acetylesterase (LgEstI) from Lactococcus garvieae
Source: PLoS One. 2023 Feb 6;18(2):e0280988. doi: 10.1371/journal.pone.0280988 (PMC9901739; doi:10.1371/journal.pone.0280988)
Supplement: S3 Fig — (DOC) [file pone.0280988.s006.doc]

**
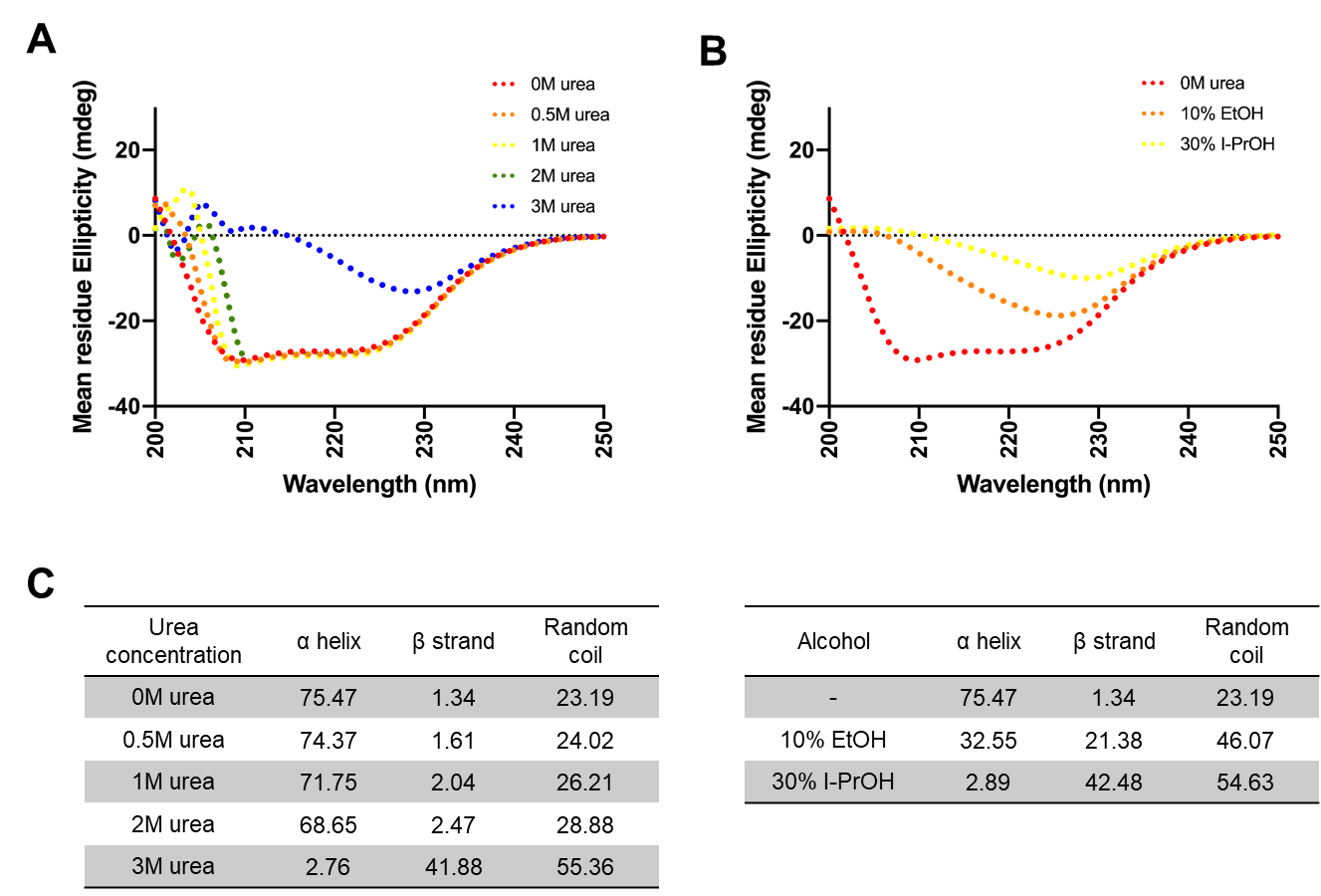
**

**Supplemental Figure S3.** Circular dichroism (CD) spectroscopy of *Lg*EstI with various denaturants. (A) CD spectra of *Lg*EstI at different concentrations of urea. (B) CD spectra of *Lg*EstI with different alcohol. Spectra were recorded at 20 °C in a 50 mM Tris-HCl (pH 8.0) and 200 mM NaCl buffer. The five spectra subtracted by baseline measured at each condition were averaged. (C) Secondary structure content (%) as determined by K2D3 software [1] from the Far-UV CD spectra as given in figures A and B.

1. Louis-Jeune C, Andrade-Navarro MA, Perez-Iratxeta C. Prediction of protein secondary structure from circular dichroism using theoretically derived spectra. Proteins Struct Funct Bioinforma. 2012;80: 374–381. doi:10.1002/prot.23188
